# Hepatitis B virus strains from Rwandan blood donors are genetically similar and form one clade within subgenotype A1

**DOI:** 10.1186/s12879-016-2149-z

**Published:** 2017-01-06

**Authors:** Theogene Twagirumugabe, Gatare Swaibu, Timothy David Walker, Magnus Lindh, Jean Bosco Gahutu, Tomas Bergström, Heléne Norder

**Affiliations:** 1 Department of Microbiology & Clinical Virology, Institute of Biomedicine at Sahlgrenska Academy, University of Gothenburg, Guldhedsgatan 10B, 41346 Gothenburg, Sweden; 2 School of Medicine and Pharmacy, College of Medicine and Health Sciences, University of Rwanda, Kigali, Rwanda; 3 Rwanda Biomedical Center-National Center for Blood and Transfusion (RBC-NCBT), Kigali, Rwanda

**Keywords:** HBV prevalence, HBV DNA, Pre-S mutants, Blood donors, Sub-Saharan Africa

## Abstract

**Background:**

Rwanda is a central African country with about 12 million inhabitants. The 1994 genocide against the Tutsi destroyed much of the infrastructure, including the health system. Although this has improved significantly, many challenges remain to be addressed. In this study, the prevalence of serological markers of past and ongoing hepatitis B virus (HBV) infection and HBV vaccine related immunity was investigated in samples from blood donors from all regions of Rwanda.

**Methods:**

The results from hepatitis B surface antigen (HBsAg) analyses of all (45,061) blood donations collected countrywide in 2014 from 13,637 first time and 31,424 repeat blood donors were compiled. Samples from 581 HBsAg negative blood donors were selected for further analysis for antibodies against HBV, anti-HBs and anti-HBc. Additional 139 samples from HBsAg positive donors were analyzed for HBeAg/anti-HBe (132 samples) and for HBV DNA. The S-gene was amplified by PCR, products sequenced, and phylogenetic analysis was performed.

**Results:**

HBsAg was found in 4.1% of first time donors with somewhat higher prevalence among those from the Central and Eastern regions than from other parts of the country. Indications of past infection was found in 21% of the HBsAg negative donors, 4.3% had only anti-HBs suggesting HBV vaccination. HBeAg was detected in 28 (21%), anti-HBe in 97 (73%), and both HBeAg and anti-HBe in 4 of 132 HBsAg positive donors. HBV DNA was found in 85 samples, and the complete S-gene was sequenced in 58 of those. Phylogenetic analysis of the sequences revealed that all HBV strains belonged to subgenotype A1, and formed one clade in the phylogenetic tree. In addition, 12 strains from first time donors had a unique 18 amino acid deletion in the N-terminal part of the pre-S2 region.

**Conclusion:**

This study indicated that the prevalence of hepatitis B is intermediate in Rwanda and that the vaccination coverage is relatively low in young adults. All surveyed Rwandan blood donors were infected with similar subgenotype A1 strains, and a high frequency of those with anti-HBe had detectable HBV DNA. Several strains had in addition a unique pre-S2 deletion, the virulence of which needs to be further studied.

**Electronic supplementary material:**

The online version of this article (doi:10.1186/s12879-016-2149-z) contains supplementary material, which is available to authorized users.

## Background

Hepatitis B virus (HBV) is a small DNA virus belonging to the *Hepadnaviridae* family [1]. Its DNA encodes for four overlapping open-reading frames, S, P, C and X [2]. The S-gene encodes for the surface proteins, preS1, preS2 and S (HBsAg); the C-gene for the nucleocapsid, and the pre-C/C region for the hepatitis B e antigen (HBeAg). The X protein is implicated in transactivation of the transcription of the HBV proteins and may play a role in carcinogenesis. The P-region encodes for a DNA-polymerase with reverse transcriptase and RNAse function, and the terminal protein, tp [1].

The viral pregenomic RNA is reverse transcribed to a partially double-stranded DNA by the viral polymerase, which by replication errors introduce mutations in the viral genome. Some of these mutations have become fixed and HBV has with time evolved into nine different genotypes (A-I) [1, 3]. Most of the genotypes are further genetically classified into subgenotypes. Genotypes A (subgenotypes A1, A3–A7), D (subgenotypes D1, D2, D4, and D6–D8) and E are prevalent in African countries [1, 4, 5].

Irrespective of genotype, early stages of HBV infection are usually asymptomatic and often unrecognized [6]. The risk for chronic infection increases inversely to the age of the patient at the moment of infection. Chronically infected patients have 15–60% lifetime risk for liver cirrhosis, liver failure and/or hepatocellular carcinoma [6–8]. Approximately 600,000 individuals die annually from complications of the infection [9, 10]. Currently, more than 2 billion people worldwide have been infected by HBV including almost 240 million with chronic infection. More than 50% of the chronically infected individuals live in Asia and in Sub-Saharan Africa where the prevalence among adults is estimated to 5–10% [4, 7].

In the natural history of chronic HBV infection, seroconversion from HBeAg to anti-HBe is a favorable sign usually accompanied by a decrease in HBV replication and remission of hepatitis [11]. However, for some patients the infecting virus strain mutate in the pre-core region to form the so-called pre-core mutant [12] or in the basal core promoter region [13, 14]. Patients infected with these mutants often have active liver disease with elevated HBV DNA levels in serum also after seroconversion to anti-HBe [15, 16]. The pre-core mutation occurs in almost all genotypes and subgenotypes of HBV, but rarely in subgenotypes A2, C1, F2, F3 and H [1, 16–18], and the basal pre-core mutations have been described from most genotypes [19, 20].

To date, there are no anti-viral compounds that completely cure HBV infection [21]. An efficacious vaccine was developed in the 1980s. Since 1991, the WHO has recommended that HBV vaccination should be included in the child vaccination program globally. Countries that have implemented this policy more than two decades ago have a substantial decline of the HBV prevalence [22]. However, the infection remains a major health burden in highly endemic zones including sub-Saharan Africa [23].

Rwanda introduced universal vaccination of children against HBV in 2003, as part of its substantial reconstruction of the infrastructure of the health system after the 1994 genocide against Tutsis. The prevalence of HBV is still significant in the adult population, and has been estimated to be about 5% based on studies on small patient groups [24–26] and on data from neighboring countries [23, 27].

The aims of this study were to evaluate the prevalence of past and current HBV infection among blood donors in all five regions of Rwanda, and to determine the genetic variability and the geographical distribution of HBV strains circulating in the country.

## Methods

### Blood donors and serum samples

**Fig. 1:**
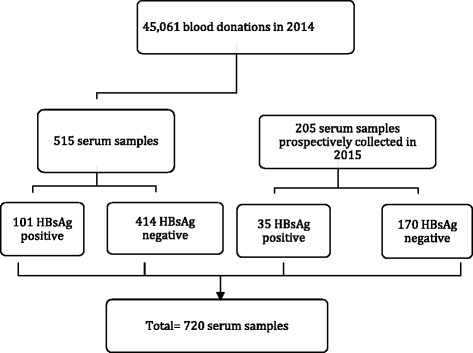
Flowchart of samples selected for further analysis in this study

Data were aggregated on analyses for HBsAg in samples from all voluntary non-remunerated first time and repeat blood donors in Rwanda recruited county-wide from five different Regional Centers for Blood and Transfusion during 2014 (Fig. 1). In total, there were 45,061 blood donors including 13,637 first time donors and 31,424 repeat donors (Table 1). All samples were routinely analyzed within 24 h after collection at the National Center for Blood and Transfusion (NCBT)-Kigali for HBsAg, anti-HCV, HIV1/2 and syphilis. The analysis for HBsAg was performed by chemiluminescent microparticles immunoassay (CMIA) using HBsAg qualitative II assay by Abbott Laboratories (Abbott Park, IL, USA) on ARCHITECT i2000SR platform according to the instructions of the manufacturer. The results of the analyses were manually transferred to a register. This register was used for selection of the samples for this study.

**Table 1:** Origin, gender and presence of HBsAg in all 45,061 blood donors in Rwanda sampled in 2014

		All blood donors			HBsAg positive blood donors		
Origin	Category of donors	Males N (%)^a^	Females N (%)^a^	Total N (%)^a^	Males N (%)^b^	Females N (%)^b^	Total N (%)^b^
Eastern Province	First time donors	961 (2.1)	516 (1.1)	1 477 (3.3)	57 (5.9)	27 (5.2)	84 (5.7)
	Repeat donors	2 314 (5.1)	547 (1.2)	2 861 (6.4)	16 (0.7)	2 (0.36)	18 (0.6)
	Subtotal	3 275 (7.3)	1 063 (2.4)	4 338 (9.6)	73 (2.2)	29 (2.7)	102 (2.4)
Kigali City	First time donors	2 326 (5.2)	1 002 (2.2)	3 328 (7.4)	138 (5.9)	37 (3.7)	175 (5.3)
	Repeat donors	13 424 (29.8)	2 727 (6)	16 151 (35.8)	6 (0.0)	2 (0.1)	8 (0.1)
	Subtotal	15 750 (35)	3 729 (8.3)	19 479 (43.2)	144 (0.9)	39 (1.0)	183 (0.9)
Northern Province	First time donors	1 618 (3.6)	523 (1.2)	2 141 (4.7)	82 (5.1)	12 (2.3)	94 (4.4)
	Repeat donors	4 550 (10.1)	392 (0.9)	4 942 (11)	2 (0.0)	1 (0.3)	3 (0.1)
	Subtotal	6 168 (13.7)	915 (2.0)	7 083 (15.7)	84 (1.4)	13 (1.4)	97 (1.4)
Southern Province	First time donors	2 144 (4.8)	1 865 (4.2)	4 009 (8.9)	90 (4.2)	29 (1.6)	119 (3.0)
	Repeat donors	3 319 (7.4)	1 753 (34.6)	5 072 (11.3)	2 (0.1)	1 (0.1)	3 (0.1)
	Subtotal	5 463 (12.1)	3 618 (3.9)	9 081 (20.2)	92 (1.7)	30 (0.8)	122 (1.3)
Western Province	First time donors	1 577 (3.5)	1 105 (2.4)	2 682 (6)	70 (4.4)	16 (1.4)	86 (3.2)
	Repeat donors	1 642 (3.6)	756 (1.7)	2 398 (5.3)	1 (0.1)	0 (0)	1 (0.0)
	Subtotal	3 219 (7.1)	1 861 (4.1)	5 080 (11.3)	71 (2.2)	16 (0.9)	87 (1.7)
Total	First time donors	8 626 (19.1)	5 011 (11.1)	13 637 (30.3)	437 (5.1)	121 (2.4)	558 (4.1)
	Repeat donors	25 249 (56.1)	6 175 (13.7)	31 424 (69.7)	27 (0.1)	6 (0.1)	33 (0.1)
	Total	33 875 (75.2)	11 186 (24.8)	45 061 (100.0)	464 (1.4)	127 (1.1)	591 (1.3)

^a^ = percentage of all donation (45 061)

^b^ = percentage of HBsAg positive of all donors of respective sex and region

All serum samples from blood donors are stored frozen at the NCBT-Kigali, and all of those with ≥ 2 mL serum from HBsAg positive first time and repeat blood donors and from about four times more HBsAg negative donors were obtained for further analyses. Since only 515 samples from donors sampled in 2014 were available (101 of those were HBsAg positive at NCBT-Kigali), an additional 205 samples from donors collected in January and February 2015 were included (35 of those were HBsAg positive at NCTB) A simplified schematic figure of the samples used for further analysis is given in Fig. 1. Only HIV negative blood donors were selected for the study.

All samples were stored at −70 °C until transportation to the Clinical Microbiology Laboratory (CML) at Sahlgrenska University Hospital, Gothenburg, Sweden, for analyses. The samples were transported on dry ice and arrived at CML within 16–18 h where they were kept at −70 °C until analyzed.

### Analysis for HBsAg, anti-HBc, anti-HBs, HBeAg, anti-HBe, and anti-HDV at CML, Sweden

At the CML, all samples were re-analyzed by CMIA for HBsAg using ARCHITECT HBsAg (Abbott Laboratories, IL,USA) on ARCHITECT i4000SR platform according to the instructions of the manufacturer.

The samples were also analyzed with CMIA for anti-HBs and total anti-HBc using ARCHITECT anti-HBs and ARCHITECT anti-HBc II (Abbott Laboratories, IL, USA), respectively on the ARTCHITECT *i*4000SR machine following the manufacturer’s instructions. HBsAg positive samples were further analyzed for HBeAg and anti-HBe with the ARCHITECT HBeAg and ARCHITECT anti-HBe (Abbott Laboratories, IL, USA) assays. All HBsAg positive and isolated anti-HBc-positive samples were analyzed for antibodies against hepatitis delta virus (anti-HDV) with the ETI-AB-DELTA K-2 kits (Diasorin, Saluggia, Italy) according to the instructions by the manufacturer.

### HBV DNA extraction

Nucleic acids were extracted from 200 μL of serum from each HBsAg and isolated anti-HBc positive sample using the NucliSens EasyMag automated extractor and kit (BioMerieux, Marcy l’Etoile, France), according to the manufacturers’ instructions. The nucleic acids were eluted in 110-μL nuclease free sterile water.

### qPCR for HBV DNA detection

The extracted nucleic acids were analyzed for HBV DNA by a TaqMan qPCR assay targeting the S region with primers HBV305S GCCAAAATTCGCAGTCCC, HBV460AS GATARTCCAGAAGAACCAAYAAGAAG and HBV-probe FAM-CGCGCGATGAGGCATAGCAGYAGGATRAARAACGCGCG-BHQ1. Each 25 μl reaction mix contained 5 μl of extracted DNA, 12.5 μl 2x universal master mix/MgCl2 and Platinum One-Step Quantitative RT-PCR System with ROX (Invitrogen, Carlsbad, CA, USA), and 0.5 μM of each primer and 0.4 μM probe. The amplification profile on Applied Biosystems 7300 platform (Carlsbad, CA, USA) was performed by initial denaturation at 95 °C for 10 min followed by 45 cycles at 95 °C for 15 s, 55 °C for 15 s and 72 °C for 30 s. RNA/DNAse-free water was used as negative control. Serial 10-fold dilutions (1/10 – 1/100 000) of two sera with 1.0 × 10^8^ and 1.2 × 10^8^ IU HBV DNA/mL, respectively, were used in each assay as positive controls and for approximate estimation of the HBV DNA concentrations of the samples investigated. The amount of HBV DNA in the positive control samples had previously been quantified by COBAS® AmpliPrep/COBAS® TaqMan® HBV Test, v2.0 (Roche AG, Basel, Switzerland). Patient samples with ct values above 42, corresponding to less than 4 IU HBV DNA/mL were considered as negative, and samples with ct values less than 23 were considered as having >5 × 10^8^ IU/mL, due to limitations in the standardization of the assay, making extrapolations uncertain. The lowest Ct values of the control samples varied from ct 25 to ct28 in the assays.

### PCR amplification of the S-gene for sequencing

A semi-nested PCR was performed to amplify the complete S-gene in a 50 μl reaction mix with 5 μl extracted nucleic acids as template. The PCR mix contained 31.9 μl of RNase-free H_2_O (Sigma), 1x Taq buffer (Applied Biosystems, Carlsbad, CA, USA), 3 mM MgCl_2_ (Applied Biosystems, Carlsbad, CA, USA), 0.2 mM dNTP, 0.3 mM of each primer, and 1 U of Taq polymerase. Pooled primers gtA1-2792S1 /gtA1-356AS1 were used for the first round PCR (Additional file 1 Table S1). The PCR reaction was performed with initial denaturation at 95 °C for 3 min followed by 40 cycles with 94 °C for 30 s, 60 °C for 60 s and 72 °C for 60 s, and one cycle at 72 °C for 10 min. Three μl of the first round product were used as template in the second amplification round with primers gtA1-2809S2 /gtA1-356AS2 (Additional file 1 Table S1) and 2.75 mM MgCl_2_. Deletions and rare mutations observed were confirmed by amplifying the complete S-gene with a semi-nested PCR using primer pools HBV-2730S and HBV-874R in the first PCR and semi-nest the product obtained in three reactions with primers HBV2730S and HBV-98R, HBV-3125S and HBV-548R, and HBV-464S and HBV-874R (Additional file 1 Table S1). The PCR reactions were performed as the PCR reaction described above, with modification of the annealing temperature to 59 °C.

### Sequencing

All amplified PCR products were purified and extracted with QIAquick PCR Purification Kit (Qiagen; Hilden, Germany) according to the manufacturer’s description. The purified products were cycle sequenced in both directions by using 1.6 μM of the same primers as in the nested PCR in the BigDye Terminator Cycle Sequencing Ready Reaction kit (Applied Biosystems) according to the manufacturer’s instructions. The sequences were obtained by the 3130 × l Genetic Analyzer (Applied Biosystems, Carlsbad, CA, USA).

### Phylogenetic analysis

The sequences obtained were analyzed in the SeqMan program in the DNAStar programme package version 10.1.2 (DNA Star Inc, Madison, WI 53705, USA). The sequences were aligned with the corresponding region of 641 sequences representing all HBV genotypes obtained from GenBank, including 252 genotype A strains from Africa. Phylogenetic analysis was carried out with the PHYLIP package version 3.65. Evolutionary distances were calculated using the F84 algorithm in the DNADIST program with a transition/transversion ratio of 1.53 with gamma correction with alpha 0.28. Phylogenetic trees were constructed using the unweight pair-group method using arithmetic averages (UPGMA) and the neighbor-joining method in the NEIGHBOR program in the PHYLIP package. Bootstrap analysis for 1,000 replicas was performed with the SEQBOOT and CONSENSE programs in the PHYLIP package.

### HBsAg subtype determination

The HBsAg subtypes of the strains were determined from the deduced amino acid sequences based on the residues at positions 122, 127 and 160 of the small S-gene [28].

### Statistical analysis

GraphPad Prism Version 6.0 g was used to compare the prevalence of HBV with origin and group of donors using the Chi-square test with Yates correction. Multivariate binary logistic regression was performed to compare the characteristics of donors with and without HBeAg adjusting for age, gender, origin, category of donors and viral load. This was also performed for donors with and without an 18 amino acid deletion in preS2. The Odds Ratio (OR) were used to express results with 95% confidence intervals (CI 95%), and p-values <0.05 were considered statistically significant. All analyses were performed using SPSS 22.0 (IBM, Chicago, USA).

## Results

There were about three times more males than females among the 45,061blood donors sampled during 2014 in five different regions of Rwanda, and most, 43%, originated from Kigali city (Table 1). The majority (70%) of the donors were repeat donors.

HBsAg was identified in sera from 558 of 13,657 (4.1%) first time donors and in 33 out of 33,424 (0.1%) repeat donors (Table 1; Fig. 1). The highest HBsAg prevalence among the first time blood donors was observed in the Eastern Province (5.3%), Kigali City (5.3%) and the Northern Province (4.4%) compared to the Southern and Western Provinces, where the HBsAg prevalence was respectively 3.0 and 3.2% (*p* <0.001, OR: 1.69 [1.42-2.02]; Table 1).

The geographical distribution of the 720 blood donors from whom samples were obtained for further analyses did not mirror the geographical distribution of all blood donors from 2014 (Table 2). There were relatively more samples obtained from the Eastern Province (*n* = 182; 25%), from where only 9.6% of all donors originated during 2014. In addition, 43% of all persons donating blood in 2014 were from Kigali City, while only 9.3% of the 720 samples obtained were from blood donors from this region (Table 2). Furthermore, about half of the samples obtained (51%) were from first time donors, while this group was represented by only 30% of all donors in 2014.

**Table 2:** Origin, gender and presence of HBsAg in 720 blood donors sampled in 2014 and 2015 and obtained for this study

		Samples obtained for study			HBsAg positive —samples used in this study		
Region	Category of donors	Males N (%)^a^	Females N (%)^a^	Total N (%)^a^	Males N (%)^b^	Females N (%)^b^	Total N (%)^b^
Eastern Province	First time donors	72 (10)	11 (1.5)	83 (11.5)	45 (6.3)	5 (0.7)	50 (7)
	Repeat donors	88 (12.2)	11 (1.5)	99 (13.8)	2 (0.3)	0 (0)	2 (0.3)
	Subtotal	160 (22.2)	22 (3.1)	182 (25.3)	47 (6.5)	5 (0.7)	52 (7.2)
Kigali City	First time donors	44 (6.1)	9 (1.3)	53 (7.4)	9 (1.3)	0 (0)	9 (1.3)
	Repeat donors	10 (1.4)	4 (0.6)	14 (1.9)	0 (−)	0 (−)	0 (0)
	Subtotal	54 (7.5)	13 (1.8)	67 (9.3)	9 (1.3)	0 (0)	9 (1.3)
Northern Province	First time donors	56 (7.8)	13 (1.8)	69 (9.6)	19 (2.6)	1 (0.1)	20 (2.8)
	Repeat donors	74 (10.3)	10 (1.4)	84 (11.7)	2 (0.3)	0 (0)	2 (0.3)
	Subtotal	130 (18.1)	23 (3.2)	153 (21.2)	21 (2.9)	1 (0.1)	22 (3.1)
Southern Province	First time donors	79 (11)	31 (4.3)	110 (15.3)	25 (3.5)	8 (1.1)	33 (4.6)
	Repeat donors	119 (16.5)	29 (4)	148 (20.6)	2 (0.3)	1 (01)	3 (0.4)
	Subtotal	198 (27.5)	60 (8.3)	258 (35.8)	27 (3.8)	9 1.3)	36 (5)
Western Province	First time donors	46 (6.4)	6 (0.8)	52 (7.2)	15 (2.1)	4 0.6)	19 (2.6)
	Repeat donors	8 (1.1)	0 (0)	8 (1.1)	1 (0.1)	0 (0)	1 (0.1)
	Subtotal	54 (7.5)	6 (0.8)	60 (8.3)	16 (2.2)	4 0.6)	20 (2.8)
Total	First time donors	297 (41.2)	70 (9.7)	367 (51)	113 (15.7)	18 (2.5)	131 (18.2)
	Repeat donors	299 (41.5)	54 (7.5)	353 (49)	7 (1)	1 (0.1)	8 (1.1)
	Total	596 (82.8)	124 (17.2)	720 (100)	120 (16.7)	19 (2.6)	139 (19.3)

^a^ = percentage of all samples obtained

^b^ = percentage of all HBsAg positive donors of respective sex and region in 2014

### Re-analysis for HBsAg

On re-analyzing 720 blood donor samples for HBsAg at CML in Sweden, nine of 136 HBsAg positive samples at NCBT in Rwanda were repeatedly non-reactive for HBsAg, and none of them were reactive for anti-HBc or anti-HBs, indicating that these samples were false positive in Rwanda. Also, there were 12 of 584 primarily HBsAg negative at NCBT that turned repeatedly reactive for HBsAg at CML. Nine of these samples were from first time donors, and all but one were positive for anti-HBc including one also positive for anti-HBs. Three of these were reactive for HBeAg and 7 for anti-HBe. The anti-HBc negative sample did not have any other marker for HBV infection, and may have had false reactivity for HBsAg at CML, which gives a false positive rate of 0.8% at CML and a 1.9% false negative rate of the test at NCBT-Kigali. The reason for this discrepancy may either be, as for two samples, a low HBsAg reactivity that was missed at NCBT in Rwanda, or a mistake in the manual transfer of the laboratory results into a register of the blood donors at NCBT.

In total there were thus 139 HBsAg positive samples and 581 HBsAg negative samples that were further analyzed for HBV markers (Table 3). Most of the HBsAg positive blood donors were first time donors and found in all age groups, however, 43.2% were younger than 26 years (Table 4).

**Table 3:** Gender and type of donor in relation to presence of HBsAg, HBeAg, anti-HBe, HBV DNA, anti-HBs and anti-HBc as markers for past infection and anti-HBs alone as indication of HBV vaccination

	First time donors			Repeat donors			All donors		
Presence of marker	Males	Females	All	Males	Females	All	Males	Females	All
HBsAg *(% of all 720 samples obtained)*	113 (15.7)	18 (2.5)	131 (18.2)	7 (1.0)	1 (0.1)	8 (1.1)	120 (16.7)	19 (2.6)	139 (19.3)
HBeAg^a^ *(% of 132 HBsAg positive samples)*	27 (20.5)	4 (3.0)	31 (23.5)	1 (0.8)	0	1 (0.8)	28 (21.2)	4 (3.0)	32 (24.2)
Anti-HBe^b^ *(% of 131 HBsAg positive samples)*	82 (62.6)	14 (10.7)	96 (73.3)	5 (3.8)	1 (0.8)	6 (4.6)	87 (66.4)	15 (11.4)	102 (77.9)
HBV DNA and HBeAg *(% of HBeAg positive samples)*	26 (96.3)	4 (100)	30 (96.8)	1 (100)	0	1 (100)	27 (96.4)	4 (100)	31 (96.9)
HBV DNA and anti-HBe *(% of anti-HBe positive samples)*	40 (48.8)	11 (78.6)	51 (53.1)	2 (40)	0	2 (33)	42 (48.3)	11 (73.3)	53 (52.0)
All with HBV DNA *(% of 139HBsAg positive samples)*	67 (48.2)	15 (10.8)	82 (59.0)	3 (2.2)	0	3 (2.2)	70 (50.4)	15 (10.8)	85 (61.2)
Anti-HBs and anti-HBc *(% of 581 HBsAg negative)*	37 (0.6)	11 (1.9)	48 (8.3)	66 (11.4)	8 (1.4)	74 (12.7)	103 (17.7)	19 (3.3)	122 (21)
Anti-HBs lacking anti-HBc *(% of 581 HBsAg negative)*	9 (1.5)	3 (0.6)	12 (2)	11 (1.9)	2 (0.3)	13 (2.2)	20 (3.4)	5 (0.9)	25 (4.3)

^a^7 samples were not sufficient for HBeAg; ^b^8 samples were not sufficient in material to run the anti-HBe

**Table 4:** Distribution of donors according to HBsAg, HBeAg, anti-HBc and anti-HBs reactivity and age groups^a^

	HBsAg		HBeAg		Anti-HBs positive		
Age group (years)	All donors	Positive N (%)	N	Positive N (%)	N	Anti-HBc positive N (%)	Anti-HBc negative N (%)
18–25	263	60 (22.8)	56	20 (35.7)	203	32 (15.8)	4 (2.0)
26–35	296	51 (17.2)	49	11 (22.4)	245	52 (21.2)	15 (6.1)
36–45	130	26 (20.0)	25	1 (4.0)	104	28 (26.9)	6 (5.8)
46–55	27	2 (7.4)	2	0 (0.0)	25	8 (32.0)	-
56–65	4	0 (0.0)	-	-	4	2 (50.0)	-
Total	720	139 (19.3)	132	32 (24.2)	581	122 (21.0)	25 (4.3)

^a^Proportions calculated with a denominator on the same row in the precedent adjacent column nN)

### HBeAg and anti-HBe

Of the 139 HBsAg positive samples, 131 (95%) could be analyzed for both HBeAg and anti-HBe, and one additional sample could only be analyzed for HBeAg (Table 3). HBeAg was found in 28 (21%) samples and anti-HBe in 97 (73%); four (3%) of the samples had both markers; and three (2.3%) lacked both HBeAg and anti-HBe (Table 3). There was a tendency of HBeAg-positive donors being younger (mean age of 25.2 +/− 6.2 years) than HBeAg-negative donors (mean age 29.8 +/− 7.6 years). This age difference was, however, not significant (OR = 0.920 [0.843-1.003], *p* = 0.059) (Additional file 1 Table S2). There was in addition no significant association between HBeAg reactivity and gender, category of donors or province of origin (Additional file 1 Table S2).

### Anti-HBc and anti-HBs

All but one of the 139 HBsAg positive samples had detectable anti-HBc. Among the 581 HBsAg negative samples, 136 (23%) had detectable anti-HBc; 122 (90%) of these had also anti-HBs (Tables 3, 4). Fourteen blood donors, all male, had isolated anti-HBc, and 25 (4.3%) had isolated anti-HBs with mean anti-HBs titers of 355 mIU/mL (range 10 - >1000 mIU/mL). The donors with anti-HBs alone originated from all regions of Rwanda and were 21–45 years old.

### HDV coinfection or superinfection

Analysis for anti-HDV was performed in 134 of the 139 HBsAg positive samples and in 11 of the 14 samples with isolated anti-HBc. All samples were negative for this marker.

### HBV DNA detection

HBV DNA was detected by qPCR in 85 (61%) of the 139 HBsAg positive samples, and in none of the 14 samples with isolated anti-HBc (Table 3). Almost all of the HBeAg positive samples (27/28; 96%) and 48/97 (49%) anti-HBe positive samples had detectable HBV DNA (Table 3). HBV DNA was also detected in all four samples with both HBeAg and anti-HBe and in none of the three samples lacking both these HBe-markers.

The HBV DNA levels, given as IU/mL based on the serial dilutions of the two positive sera were higher in HBeAg than anti-HBe positive sera with median 3.7 × 10^6^ vs. 500 IU HBV DNA /mL (*p* <0.0001). The four samples with both HBe-markers had a median value of 4.3 × 10^3^ IU HBV DNA /ml. There were 31 samples with a viral load of ≥10^4^ IU HBV DNA /ml (Additional file 1 Table S2); 22 of these samples had detectable HBeAg (Additional file 1 Table S2).

### Sequencing the S-gene

The complete S-gene could be amplified and sequenced in 58 of the 85 HBV DNA-positive samples. Based on the deduced amino acid sequence of the small S-gene, the subtype was inferred to *adw2* for 56 strains (97%), since they expressed Lys^122^, Pro^127^ and Lys^160^. Two samples, RW14-173 and RW14-203, expressed Arg^122^ and were *ayw1* strains. Both these two later strains were from first time donors, one from the Eastern and the other from the Western Province. None of the sequenced strains had the vaccine escape mutation Gly145/Arg/Ala.

Several mutations were observed when the pre-S regions were analyzed. The most common mutations were deletion in the pre-S1 and pre-S2 regions. Two larger deletions in pre-S1 was identified in two strains, RW14-219 with a deletion between amino acids 30 and 84, and RW14-226 with a 20 amino acid deletion between residues 67 and 86. There were in addition an 18 amino acid deletions in the N-terminal region of pre-S2, between residues 5 and 22 in twelve strains (RW14-03, − 05, −10, −14, −40, −41, −43, −118, −136, −173, −190, and −216). Other deletions (9, 11 and 14 amino acids) in the same region were observed for another three strains (RW14-22, −198 and −222). The18 amino acid deletion in pre-S2 was not independently associated with age or gender of the donors, presence of HBeAg/anti-HBe, or viral load (Additional file 1 Table S3).

### Phylogenetic analysis

**Fig. 2:**
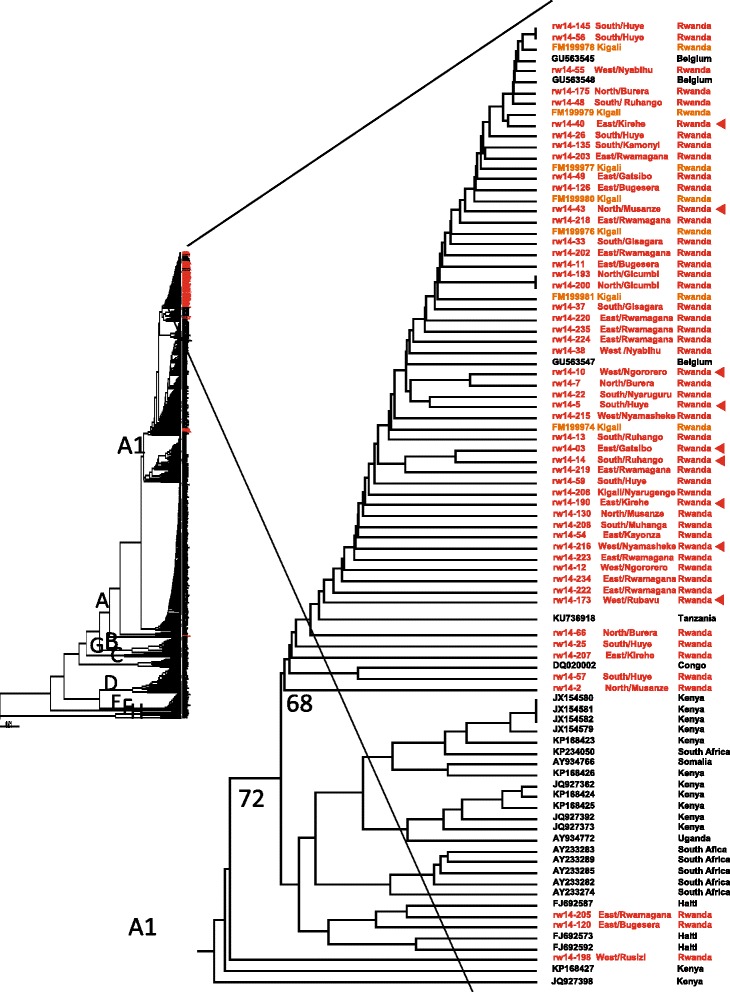
Phylogenetic tree based on 1212 nucleotides of the large S-gene (pre-S1/pre-S2/S) of 699 strains representing all HBV genotypes. All 252 available genotype A sequences of the large S-gene of strains from different African countries were included in the analysis. The genotypes are indicated on the branches. The branch with 53 of the 58 A1 strains from this study and additional 7 strains from Rwanda and 21 strains from other African countries is enlarged. The strains sequenced in this study are shown in red. Strains obtained from GenBank are given with accession number and country of origin at the nodes. Strains with the 18 amino acid deletion in pre-S2 are marked with a red arrowhead at the node

Based on phylogenetic analysis of the complete S-gene, all 58 sequenced strains in this study were found to belong to subgenotype A1 (Fig. 2). All strains belonging to this subgenotype formed three subclades in the phylogenetic tree. All but five of the strains in this study formed one of these subclades together with previously published strains from Kigali, Rwanda, and one strain each from Somalia and the Democratic Republic of the Congo (Fig. 2). The formation of this clade was supported by 68% bootstra*p* value. Four of the strains from the blood donors in Rwanda were not found in this A1 subclade, and were more closely related to Indian, Chinese and Japanese subgenotype A1 isolates. Another strain, RW14-41 from a first time donor in the Eastern Province, was divergent and formed a separate branch within genotype A. This strain had in addition to the large 18 amino acid deletion in pre-S2 also several amino acid substitutions in pre-S2, and was also divergent from the genotype A strains when the small S-gene was analyzed.

The subgenotype A1 strains also formed separate clades when the small S-gene was analyzed. In the clade formed by the small S-genes of most strains from Rwanda, there was one branch formed mainly by strains from Rwanda together with 6 strains from Kenya. The other strains from Rwanda were intermixed with A1 strains from other African countries in this clade (Additional file 2 Figure S1).

## Discussion

This study on HBV infections in blood donors from all five different provinces of Rwanda confirms that this country has intermediate endemicity for this infection, despite its geographical location in a region of Africa with high HBV prevalence. The prevalence of HBsAg positivity was equal in the five regions of Rwanda, and was 4.1% among first time blood donors. The actual HBsAg prevalence may differ between the regions, since the number of first time blood donors investigated from each region did not mirror the population size in the regions, especially not from Kigali City. In addition, fist time donors may not represent the general population, especially since all HIV positive blood donors were excluded in the study. However, the HBsAg prevalence found in this study is comparable to that previously described from different groups of female patients and health care workers in Kigali City [23–26], which indicates that the results obtained from the first time blood donors may be regarded as indicative of the HBV prevalence in the general population.

All Rwandan children are vaccinated against HBV, starting from six-weeks of age, since 2003, when the pentavalent vaccine was introduced by the WHO-sponsored Expanded Program of Immunization through Gavi, the Vaccine Alliance. Thus, most HBV infections are now occurring among adults. In this study, about one quarter (23%) of the HBsAg negative blood donors, who may be representative of the wider adult population in the country, have been exposed to HBV and cleared the infection. This is a relatively lower prevalence of anti-HBc and anti-HBs compared to many other Sub-Saharan African countries especially those in West Africa [29], which suggests that a large part of the Rwandan adult population is susceptible to hepatitis B. In addition, the vaccination coverage seems to be low among adults in Rwanda, with 4.3% of the blood donors in this study having only anti-HBs, indicating immunity obtained by HBV vaccination. This is in accordance with a study on 378 health care workers in Rwanda, among who 4.5% reported having received the HBV vaccine [24]. There is thus a need to implement a vaccination policy for adults in Rwanda to reduce the number of new infections in this predominantly sexually active population.

In Africa, HBV genotypes A, D and E are the most prevalent [30]. The findings in this study were consistent with this, with all Rwandan strains belonging to genotype A. The strains formed a separate clade among the subgenotype A1 strains in the phylogenetic tree, and strains from the five different regions of the country were intermixed with each other within this clade. This may indicate that once there was an introduction of this strain which has spread throughout the country, and is now the dominant HBV strain. The absence of strains of other HBV genotypes in samples from the blood donors is surprising, and may indicate that hepatitis B was rather recently introduced into the country. This finding is consistent with the identified intermediate to low prevalence of HBV among blood donors, and the single major clade with a common ancestor formed by the strains from all regions of the country. Genotype D has been reported from neighboring countries and from pregnant HIV positive women in Kigali City, however the country of origin of these women was not reported [31, 32]. In this study there were few serum samples from HBsAg positive blood donors from Kigali City, therefore other HBV genotypes may be present in the capital of Rwanda, even if they were not identified in this study. However, other previously described HBV strain from Kigali were of subgenotype A1 [31] and was found intermixed with strains from this study in the A1 clade that was formed only by Rwandan strains in the phylogenetic tree. A comprehensive study on liver disease patients recruited from different regions of the country including more patients from Kigali City may be able to determine if A1, and especially this strain forming the A1 clade, is predominant in all HBV carriers of Rwandan nationality.

Several of the HBV strains sequenced in this study had a large deletion in the N-terminal region of pre-S2. These strains were interspread in the A1 clade, which indicates that the deletion had occurred independently in the strains. There was no difference between the carriers of strains with and without this deletion with regard to age, gender, origin, viral load, or HBeAg. The liver disease progression of the blood donors infected with strains with and without the deletion mutant could not be determined in this study. The impact on disease progression of these strains may be investigated by comparing their prevalence in chronic HBV carriers and patients with HBV-induced HCC and/or liver cirrhosis in Rwanda, since such deletions have been found in patients infected by other genotypes and have developed cirrhosis or HCC [33–36].

Complete genomes of A1 strains with and without the deletion will reveal if this mutation predisposes for other mutations in the HBV genome as in the promoter regions. Sequencing strains from HBsAg positive liver disease patients from different regions of Rwanda will also reveal if this strain is more virulent or more easily transmissible than the other A1 strains found in this study. In addition, more than half of the blood donors infected with anti-HBe positive strains also had detectable HBV DNA, indicating a high prevalence of the pre-core mutant or basal core promoter mutants [37, 38]. The HBV pre-core mutant strains have been shown to cause fulminant hepatitis in the recipient when horizontally transmitted [39–41] and found in both chronic active and inactive hepatitis HBsAg carriers [42]. In addition, the basal core promoter mutants have been shown to be involved in the development of cirrhosis and HCC [43–46]. The mutations in the Rwandan strains and their involvement in liver disease progression in HBV carriers could not be evaluated in this study, and need to be investigated. Further studies are therefore needed on HBV strains from patients with different stages of liver disease in Rwanda.

## Conclusions

This is the first study to show an extensive prevalence of HBV serum markers among adults in Rwanda. Furthermore, the study showed that all blood donor strains belonged to subgenotype A1, and that there is a high prevalence of HBV strains with several pre-S mutations whose clinical implications need to be explored. These data are of paramount importance for healthcare planning for management, vaccination policy, and surveillance of HBV infections in Rwanda.

## Additional files

Additional file 1: Table S1. List of primers used to sequence the S-gene. **Table S2** Distribution of blood donors according to the HBeAg reactivity and associated factors. **Table S3** Factors associated with the 18 amino acid preS2 deletions compared to strains without. (DOCX 24 kb)
Additional file 2: Figure S1. Phylogenetic tree of the small S-gene encoding for HBsAg of 527 strains. The branch with 52 of the 58 A1 strains from this study and additional 7 strains from Rwanda and 13 strains from other African countries is enlarged. The strains sequenced in this study are shown in red. Strains obtained from GenBank are given with accession number and country of origin at the nodes. Strains with an 18 amino acid deletion in preS2 are marked with a red arrowhead at the nodes. (PPTX 93 kb)

## Abbreviations

Anti-HBc: Hepatitis B viral core antibodies
Anti-HBe: Hepatitis B viral e antibodies
Anti-HBs: Hepatitis B viral surface antibodies
CI: Confidence interval
CMIA: Chemiluminescent microparticles immunoassay
CML: Clinical microbiology laboratory, gothenburg, Sweden
DNA: Deoxyribonucleic acid
HBeAg: Hepatitis B viral e antigen
HBsAg: Hepatitis B viral surface antigen
HBV: Hepatitis B virus
HCC: Hepatocellular carcinoma
HCV: Hepatitis C virus
HIV: Human immunodeficiency virus
NCBT: National center for blood transfusion
OR: Odds ratio
PCR: Polymerase chain reaction
RBC: Rwanda biomedical center
RNA: Ribonucleic acid
WHO: World Health Organization
