# Supplementary material for: Hepatitis B virus strains from Rwandan blood donors are genetically similar and form one clade within subgenotype A1
Source: BMC Infect Dis. 2017 Jan 6;17:32. doi: 10.1186/s12879-016-2149-z (PMC5217631; doi:10.1186/s12879-016-2149-z)
Supplement: Additional file 1: Table S1. — List of primers used to sequence the S-gene. Table S2 Distribution of blood donors according to the HBeAg reactivity and associated factors. Table S3 Factors associated with the 18 amino acid preS2 deletions compared to strains without. (DOCX 24 kb) [file 12879_2016_2149_MOESM1_ESM.docx]

- **SUPPLEMENTAL MATERIAL**
- Table S1. Primers used for amplification of the S-gene

| - Primer designation | - Primer sequence |
| --- | --- |
| - gtA1-2792S1 | - GGAAACTACACGTAGCGCCTCATTTTG |
| - gtA1-2792S2 | - GGAAACTACACGTAGCGCTTCATTTTG |
| - gtA1-356AS1 | - ATTTGTCCTGGTTATCGCTGGATGTGT |
| - gtA1-356AS2 | - ATTTGTCCTGGTTATCGCTGGATGTGT |
| - gtA1-2809S1 | - CCTCATTTTGCGGGTCACCATATTCTT |
| - gtA1-2809S2 | - CCTCATTTTGCGGGTCACCATACTCTT |
| - gtA1-2809S3 | - CTTCATTTTGCGGGTCACCATATTCTT |
| - HBV-2730S1 | - CATTACTYCCAGACCCGACATTATTTACAT |
| - HBV-2730S2 | - CATTACTTCCAGACCCGACACTATTTACAT |
| - HBV-2730S3 | - CATTACTTCCAGACCCGACATTATTTGCAT |
| - HBV-2730S4 | - CATTACTTCCAGACCCGACATTAATTACAT |
| - HBV-2730S5 | - CATTACTCCCAGACCCGACCTTATTAACAT |
| - HBV-2730S6 | - CATTACTTCCAAACCCGACATTATTTACAT |
| - HBV-2730S7 | - CATGAGGCCCAGACCCGACCTTATATACAT |
| - HBV-2730S8 | - CATTACTTCCAGACTCGACATTATTTACAT |
| - HBV-98R1 | - TTGACGAGATGTGAGAGGCARTATT |
| - HBV-98R2 | - TTGACGATATGTGAGAGACAATATT |
| - HBV-98R3 | - TTGACGATATGTGAGAGGCAATATT |
| - HBV-98R4 | - TGGAGGAGATGGGAGAGGCAATATT |
| - HBV-3125S1 | - AGTCAGGAAGGCMGCCTACTCCCAT |
| - HBV-3125S2 | - AGACAGGGAGGCAGCCTACTCCCAT |
| - HBV-3125S3 | - CCTATCGAAGGCAGCCTACTCCCAT |
| - HBV-3125S4 | - AGGCAGGAAGGCAGCCTACTCCCAT |
| - HBV-3125S5 | - AGTCACGTAGGCAGCCTACTCSCAT |
| - HBV-3125S6 | - AGTCAAGGAGGCAGCCCACTCCCAT |
| - HBV-464S1 | - TTGCCCGTTTGTCCTCTAATTCCAGGATCC |
| - HBV-464S2 | - TTGCCCGTTTGTCCTCTSATTCCAGGATCC |
| - HBV-874R1 | - ACCCCAACTTCCAATTACATAKCCCATGAA |
| - HBV-874R2 | - RCCCCAACTTCCAATTATRTATCCCATGAA |
| - HBV-874R3 | - ACCCCTACTTCAAATTACRTATCCCaTGAA |
| - HBV-874R4 | - GCCCCAaCTTCCAATYACATATCCcATGAA |
| - HBV-548R1 | - TTTTGTACAGCAACATGRGGGAAACATAGA |
| - HBV-548R2 | - TTTTGTACAGCAACATGAGGGAATCATAGA |

Table S2. Distribution of donors according to the HBeAg reactivity and associated factors

| - **Characteristics** | - **HBeAg positive** - **(N=32)** | - **HBeAg negative** - **(N=100)** | - **Adjusted OR or mean difference (95%CI)** | - **p-Value** |
| --- | --- | --- | --- | --- |
| - Age (years); mean+/-SD | - 25.16+/-6.24 | - 29.80+/-7.59 | - 0.920 (0.843 – 1.003) | - 0.059 |
| - Gender:   Females  Males | - 4 (22.2%) - 28 (24.6%) | - 14 (77.2%) - 86 (75.4%) | - 0.176 (0.023 – 1.372) - 1 | - 0.097 |
| - Category of donors:   Repeat  First time   - Residence:   South  East  Kigali  North  West | - 1 (14.3%) - 31 (25.0%) - 13 (36.1%) - 10 (21.7%) - 1 (11.1%) - 3 (13.6%) - 5 (26.3%) | - 7 (85.7%) - 93 (75.0%) - 23 (63.9%) - 36 (78.3%) - 8 (88.9%) - 19 (86.4%) - 14 (73.7%) | - 1.065 (0.103 –10.969) - 1 - 1.119 (0.259 – 4.826) - 0.197 (0.036 – 1.095) - 0.153 (0.009 – 2.568) - 0.186 (0.023 – 1.484) - 1 | - 0.958 - 0.880 - 0.063 - 0.192 - 0.112 |
| HBV DNA (IU/ml)  <10  10-100  100-1000  1000-10000  10000-100000  >100000 | 1 (1.8%)  1 (7.7%)  4 (19%)  4 (40%)  2 (40%)  20 (76.9%) | 56 (98.2%)  12 (92.3%)  17 (81%)  6 (60%)  3 (60%)  6 (23.1%) | 1*  35.502 (9.101-138.494) | <0.0001 |

Hosmer & Lemeshow Test (*Chi-square*: 3.878; *df:* 8; *p-value*: 0.868)

* In this model, category with >100000 IU/ml was compared to the rest. The higher viral load was independently associated with HBeAg positivity.

Table S3. Factors associated with the 18 amino acid preS2 deletions compared to strains without

|  | **PreS2 deletion** | | | |
| --- | --- | --- | --- | --- |
| **Variable** | **Absent**  **(N=42*; 77.8%)** | **Present**  **(N=12; 22.2%)** | **OR (95%CI)** | **p-value** |
|  |  |  |  |  |
| *Age in years (mean+/-SD)* | 25.57 +/- 6.89 | 28.50+/-5.47 | *1.121 (0.986-1.275)* | *0.081* |
| *Gender* |  |  |  |  |
| Male; n (%) | 37 (78.7%) | 10 (21.3%) | *1* |  |
| Female; n (%) | 5 (71.4%) | 2 (28.6%) | *1.668(0.123-22.545)* | *0.700* |
| *Residence* |  |  |  |  |
| East; n (%) | 18 (78.3%) | 5 (21.7%) | *0.434(0.012-16.160)* | *0.651* |
| Kigali; n (%) | 1 (50%) | 1 (50%) | *-* | *-* |
| North; n (%) | 7 (87.5%) | 1 (12.5%) | *0.160 (0.003-9.126)* | *0.375* |
| South; n (%) | 12 (85.7%) | 2 (14.3%) | *0.255(0.007-8.894)* | *0.451* |
| West; n (%) | 4 (57.1%) | 3 (42.9%) | *1* |  |
| *Category of donors* |  |  |  |  |
| First time; n (%) | 41 (77.4%) | 12 (22.6%) | *1* |  |
| Repeat; n (%) | 1 (100%) | 0 (0%) | *1.842* | *-* |
| *HBeAg* |  |  |  |  |
| Positive; n (%) | 21 (75%) | 7 (25%) | *1* |  |
| Negative; n (%) | 19 (86.4%) | 3 (13.6%) | *0.427(0.051-3.543)* | *0.430* |
| *HBV-DNA level (IU/ml)*  <10  10-100  100-1000  1000-10000  10000-100000  >100000 | 2 (100%)  4 (100%)  7 (77.8%)  5 (50%)  4 (80%)  20 (76.9%) | 0 (0%)  0 (0%)  0 (0%)  5 (50%)  1 (20%)  6(23.1%) | *-*  *-*  *-*  *2.080 (0.118-36.757)*  *1.080 (0.051-22.883)*  *1* | *-*  *-*  *-*  *0.617*  *0.961* |

*Hosmer & lemeshow Test (Chi-Square=9.095 df=8, p-value=0.334)*

* Four (4) samples with other smaller deletions were excluded from the analysis

No independent factors associated with occurrence of Pre-S2 large deletions in this model

- **Figure legend**
- Figure S1. Phylogenetic tree based on 681 nucleotides of the small S-gene encoding for HBsAg of 527 strains. The branch with 52 of the 58 A1 strains from this study and additional 7 strains from Rwanda and 13 strains from other African countries is enlarged. The strains sequenced in this study are shown in red. Strains obtained from GenBank are given with accession number and country of origin at the nodes. Strains with an 18amino acid deletion in preS2 are marked with a red arrowhead at the nodes.
