# Supplementary material for: Hepatitis B virus strains from Rwandan blood donors are genetically similar and form one clade within subgenotype A1
Source: BMC Infect Dis. 2017 Jan 6;17:32. doi: 10.1186/s12879-016-2149-z (PMC5217631; doi:10.1186/s12879-016-2149-z)
Supplement: Additional file 2: Figure S1. — Phylogenetic tree of the small S-gene encoding for HBsAg of 527 strains. The branch with 52 of the 58 A1 strains from this study and additional 7 strains from Rwanda and 13 strains from other African countries is enlarged. The strains sequenced in this study are shown in red. Strains obtained from GenBank are given with accession number and country of origin at the nodes. Strains with an 18 amino acid deletion in preS2 are marked with a red arrowhead at the nodes. (PPTX 93 kb) [file 12879_2016_2149_MOESM2_ESM.pptx]

## Slide 1
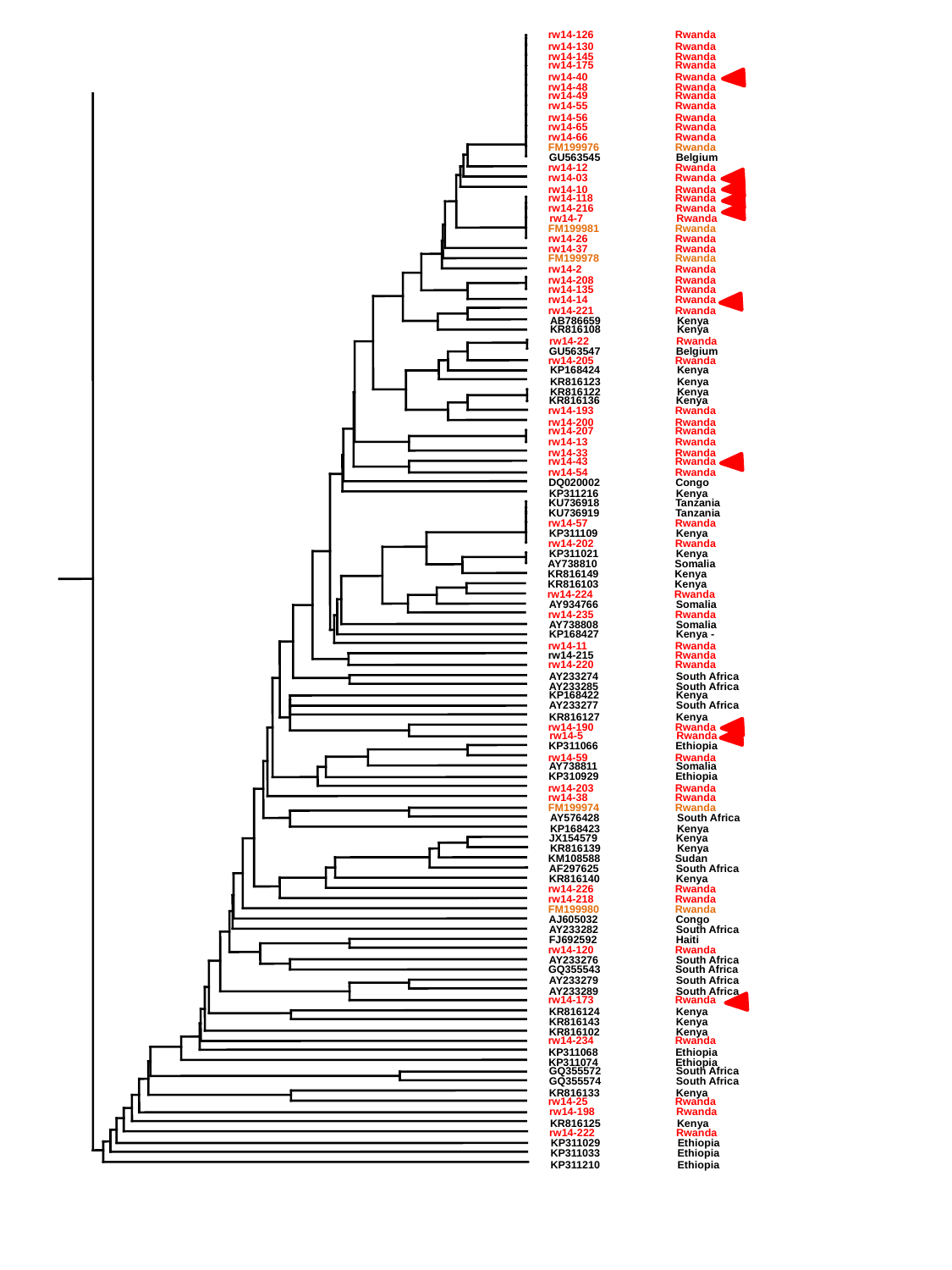

rw14-126	Rwanda
rw14-130	Rwanda
rw14-145	Rwanda
rw14-175	Rwanda
rw14-40	Rwanda
rw14-48	Rwanda
rw14-49	Rwanda
rw14-55	Rwanda
rw14-56	Rwanda
rw14-65	Rwanda
rw14-66	Rwanda
FM199976	Rwanda
GU563545 	Belgium
rw14-12	Rwanda
rw14-03	Rwanda
rw14-10	Rwanda
rw14-118	Rwanda
rw14-216	Rwanda
rw14-7	Rwanda
FM199981	Rwanda
rw14-26	Rwanda
rw14-37	Rwanda
FM199978	Rwanda
rw14-2	Rwanda
rw14-208	Rwanda
rw14-135	Rwanda
rw14-14	Rwanda
rw14-221	Rwanda
AB786659	Kenya
KR816108 	Kenya
rw14-22	Rwanda
GU563547	Belgium
rw14-205	Rwanda
KP168424	Kenya
KR816123 	Kenya
KR816122 	Kenya
KR816136 	Kenya
rw14-193	Rwanda
rw14-200	Rwanda
rw14-207	Rwanda
rw14-13	Rwanda
rw14-33	Rwanda
rw14-43	Rwanda
rw14-54	Rwanda
DQ020002	Congo
KP311216 	Kenya
KU736918	Tanzania
KU736919	Tanzania
rw14-57	Rwanda
KP311109	Kenya
rw14-202	Rwanda
KP311021	Kenya
AY738810	Somalia
KR816149	Kenya
KR816103	Kenya
rw14-224	Rwanda
AY934766	Somalia
rw14-235	Rwanda
AY738808	Somalia
KP168427	Kenya -
rw14-11	Rwanda
rw14-215	Rwanda
rw14-220	Rwanda
AY233274	South Africa
AY233285	South Africa
KP168422	Kenya
AY233277	South Africa
KR816127	Kenya
rw14-190	Rwanda
rw14-5	Rwanda
KP311066	Ethiopia
rw14-59	Rwanda
AY738811	Somalia
KP310929	Ethiopia
rw14-203	Rwanda
rw14-38	Rwanda
FM199974	Rwanda
AY576428	South Africa
KP168423	Kenya
JX154579	Kenya
KR816139	Kenya
KM108588	Sudan
AF297625	South Africa
KR816140	Kenya
rw14-226	Rwanda
rw14-218	Rwanda
FM199980	Rwanda
AJ605032	Congo
AY233282	South Africa
FJ692592	Haiti
rw14-120	Rwanda
AY233276	South Africa
GQ355543	South Africa
AY233279	South Africa
AY233289	South Africa
rw14-173	Rwanda
KR816124	Kenya
KR816143	Kenya
KR816102	Kenya
rw14-234	Rwanda
KP311068	Ethiopia
KP311074	Ethiopia
GQ355572	South Africa
GQ355574	South Africa
KR816133	Kenya
rw14-25	Rwanda
rw14-198	Rwanda
KR816125	Kenya
rw14-222	Rwanda
KP311029	Ethiopia
KP311033	Ethiopia
KP311210	Ethiopia
